# Pathobiological Interactions of Local Bone Marrow Renin-Angiotensin System and Central Nervous System in Systemic Arterial Hypertension

**DOI:** 10.3389/fendo.2020.00425

**Published:** 2020-08-07

**Authors:** Rafiye Ciftciler, Ibrahim Celalettin Haznedaroglu

**Affiliations:** Department of Hematology, Faculty of Medicine, Hacettepe University, Ankara, Turkey

**Keywords:** renin-angiotensin system, bone marrow, atherosclerosis, hypertension, central nervous system

## Abstract

Circulating renin-angiotensin system (RAS) and local paracrin-autocrin-intracrin tissue-based RAS participate in numerous pathobiological events. Pro-inflammatory, pro-fibrotic, and pro-thrombotic consequences associated with local RAS activation have been detected at cellular and molecular level. Regenerative progenitor cell therapy in response to RAS modulating pharmacotherapy has emerged as an adjunct in the context of endothelial cell injury and regeneration to improve regeneration of the vascular endothelium. Local hematopoietic bone marrow (BM) RAS symbolizes the place of cross-interaction between vascular biology and cellular events from embryogenesis to definitive hematopoiesis underlying vascular atherosclerosis. The BM microenvironment also contains Mas receptors, which control the proliferative role of Ang 1-7 on hematopoietic stem cells. Ang 1-7 is produced from Ang-II or Ang-I with the help of ACE2. Various tissues and organs also have an effect on the RAS system. The leukocytes contain and synthesize immunoreactive angiotensinogen species capable of producing angiotensin in the basal state or after incubation with renin. The significance of RAS employment in atherosclerosis and hypertension was indicated by novel bidirectional Central Nervous System (CNS) RAS–BM RAS communications. Myeloid cells generated within the context of hematopoietic BM RAS are considered as the initiators and decision shapers in atherosclerosis. Macrophages in the atherosclerotic lesions contain angiotensin peptides by which RAS blockers inhibit monocyte activation and adherence. Furthermore, vascular biology in relation to inflammation and neoplasia is also affected by local tissue RAS. The purpose of this article is to outline interactions of circulating and local angiotensin systems, especially local bone marrow RAS, in the vascular pathobiological microenvironment of CNS.

## Introduction

As previously known, renin-angiotensin system (RAS) was described as an endocrine system that regulates blood pressure and body electrolyte balance (1). Recently RAS, which is considered as an “ubiquitous” system with various effects on tissue physiology and homeostasis, has been shown to be locally expressed in different tissues (2). With the detection of novel RAS components, the emerging tissue RAS concept has expanded the physiological and clinical location of the RAS (2). Circulating local RAS and local paracrin-autocrin-intracrin tissue-based RAS participate in numerous pathobiological events. Pro-inflammatory, pro-fibrotic, and pro-thrombotic consequences associated with local RAS activation have been detected at cellular and molecular level (2). For these reasons, it is important to know RAS components, tissue-specific expressions of RAS and how they can change under pathological circumstances (2). There is a RAS located in the bone marrow (BM) microenvironment and within the hematopoietic stem cells (HSC). The concept of local BM RAS, which is active in primitive and definitive hematopoiesis, had been proposed by Haznedaroglu and coworkers about three decades ago (3, 4).

RAS molecules, particularly ACE, ACE2, AGT, AGTR1, AGTR2, AKR1C4, AKR1D1, ANPEP, ATP6AP2, CMA1, CPA3, CTSA, CTSD, CTSG, CYP11A1, CYP11B1, CYP11B2, CYP17A1, CYP21A2, DPP3, EGFR, ENPEP, GPER, HSD11B1, HSD11B2, IGF2R, KLK1, LNPEP, MAS1, MME, NR3C1, NR3C2, PREP, REN, RNPEP, THOP1 are available in the BM (5). These molecules affect the entire stem cell-oriented hematopoiesis. Local BM RAS seems to be the major station of cross-talk between hematopoietic stem/progenitor cells and their afferent functions within the heart, kidney and Central Nervous System (CNS) as a (patho)biological trigger for vascular atherosclerosis (6). There is also a local CNS RAS. The importance of RAS and BM-RAS activity in atherosclerosis and hypertension was indicated by novel bidirectional CNS RAS–BM RAS communications (7–9). The purpose of this article is to outline interactions of circulating and local angiotensin systems, especially local bone marrow RAS, in the vascular pathobiological microenvironment of CNS.

## Local Bone Marrow Renin Angiotensin System: Definition and a Brief History

Major RAS molecules, including renin, angiotensinogen, angiotensin receptors and ACE, are all found in the BM microenvironment (10). Haznedaroglu et al. first proposed the idea of a local hematopoietic RAS in BM in 1996. The study supposed that there is a locally active RAS in the BM that affects the production, proliferation growth, and differentiation of hematopoietic cells (3). Later, evidence of a local RAS in the BM increased day by day. Locally active BM RAS influences important pathways in physiological and pathological blood cell production by autocrine, paracrine and intracrine routes (11, 12). The development of hematopoietic niche, erythropoiesis, myelopoiesis, thrombopoiesis and other cellular linage is controlled by local BM RAS peptides (13–16). Additionally, many important pathobiological events such as cellular proliferative events, mobilization, angiogenesis, fibrosis, and apoptosis in the cytokine network are affected by RAS molecules (11, 12, 17, 18). Local RAS in the BM stromal niche controls important hematopoietic functions (10, 12–15, 19, 20). The BM stromal microenvironment contains important peptides that are components of the RAS (11, 12).

BM stromal microenvironment includes AT1R and AT2R (angiotensin type 1 and type 2 receptors, respectively) and inhibitory tetrapeptide AcSDKP (N-acetyl-Ser-Asp-Lys-Pro) (11, 12). The major RAS effector agent angiotensin II (Ang II) performs its impacts on the hematopoietic system by activating the AT1Rs and AT2Rs, along with the BM microenvironment (11, 12). As a result of ACE's (CD143) disrupting the inhibitory tetrapetide, AcSDKP, priming of stem cells into S-phase is triggered (16, 21). Additionally, Ang-II stimulates the AT1/AT2 receptors, so it has stimulating or inhibitory effects on erythropoietin, thrombopoietin and other hematopoietic cytokines in normal hematopoiesis and myeloproliferative diseases (16, 22, 23). Multiple clinical studies have been made to evaluate the role of local BM RAS in several diseases (18, 24–30).

Phase I/II clinical trials of a pharmaceutical agent of peptide angiotensin 1-7 (Ang-1-7) have been directed to assess the role of local BM RAS in different diseases (18, 24, 25). Other participating components of Ang II and RAS such as Ang IV [Ang-(3-8)] and Ang-(1-7) play a regulatory role on the cardiovascular system (26). Moreover, ACE2 and Mas receptor are very important components of the option line of RAS and are expressed in Hematopoietic stem/progenitor cells (HSPCs) (27). Activation of the ACE2/Ang-(1-7)/MasR axis stimulates the functions of HSPCs related to vascular repair and repulses dysfunctions caused by chronic pathological situations (27). On the other hand, white blood cells locally produce angiotensin peptides (28–30). Gomez et al. showed that circulating rat leukocytes express the angiotensinogen gene. It showed that leukocytes contain and synthesize immunoreactive angiotensinogen species that can produce angiotensin in the basal state or after incubation with renin. As a system that produces angiotensin, leukocytes can be important in modulating inflammatory responses, tissue damage, and cardiovascular pathology such as hypertension (28).

The supposition that local autocrine BM RAS may be effective in neoplastic hematopoiesis supports the prominent functions of local RAS in primitive embryonic hematopoiesis (31–33). Likewise the considerable functions of local RAS, which are involved in primitive embryonic hematopoiesis, also reinforce the supposition that local autocrine BM RAS may have an effective role in neoplastic hematopoiesis (19). Critical RAS modulating agents such as renin, ACE, angiotensinogen, and AngII have been previously described in leukemic malignant cells (34–36). Recently, Yamashita et al. showed that angiotensin-(1-12) generation is revealed in the BM of rats. Chymase-mediated Ang II production in BM was importantly higher than ACE-mediated and 280-fold higher than that in the heart (37). CD68 positive myeloid lineage cells, especially myeloid progenitors, have higher chymase expression than CD68 negative lymphoid lineage cells in BM (37).

## Local Bone Marrow Renin Angiotensin System and Atherosclerosis

**Figure 1:**
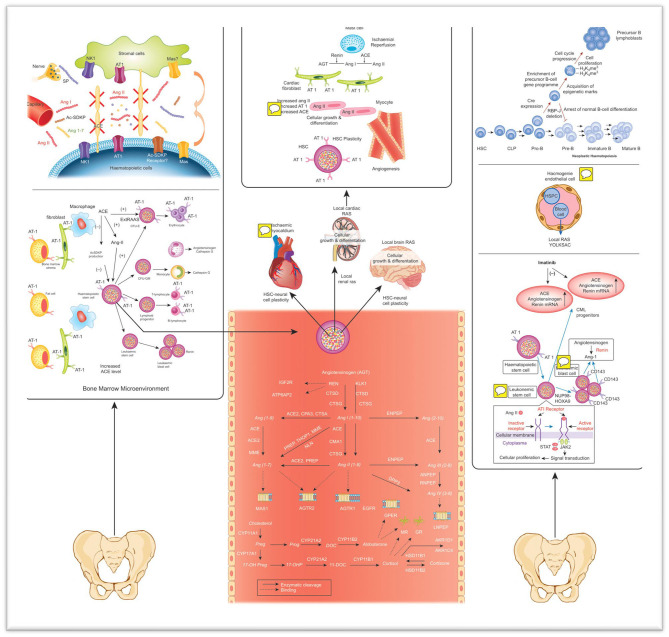
The effect of local RAS on normal and leukemic hematopoiesis, as well as plasticity and systemic circulation, are shown (19). Ang I, Ang II, Ac-SDKP, and Ang 1-7 are secreted in different tissues and transferred to the BM through circulation. SP is secreted from the nerve end protruding into the BM. ACE converts Ang II from Ang I and reduces bioactive SP, Ac-SDKP and Ang 1-7 (38). Mas, with the Ang 1-7 receptor, is detected in the BM. Peptides in RAS can stimulate hematopoietic cells (39). Furthermore, local BM RAS has a function in the development, formation, proliferation, and differentiation of hematopoietic progenitors. Angiotensin peptides and steroid hormones were symbolized in gray. Lymphoid cells cannot differentiate throughout the normal B-cell pathway (40). Therefore, the leader B-cell gene undergoes epigenetic initiation and enrichment of a precursor B-cell gene programme. As a result, cell cycle progression and cell proliferation are observed in mutant cells. This leads to the enlargement of a lymphoblast population and the development of leading B cell leukemia (5, 19, 23, 38, 40, 41). HSPC, Hematopoietic stem/progenitor cells; ACE, Angiotensin converting enzyme; CML, Chronic myeloid leukemia; Ang, Angiotensinogen; CFU-GM/E, Colonyforming units-Granulocyte-Macrophage/erythroid; JAS/STAT, Janus kinase/Signal transduction and transcription; BC, Blood cell; Ang, Angiotensin; Preg, Pregnanolone; Prog, Progesterone; DOC, deoxycortisol; 17-OHP, 17-OH Progesterone; ACE, angiotensin I converting enzyme; ACE2, angiotensin I converting enzyme type 2; AGTR1, angiotensin II type 1 receptor; AGTR2, angiotensin II type 2 receptor; AKRIC4, aldo-ketoreductase family 1 member C4; AKRID1, aldo-ketoreductase family 1 member D1; ANPEP, alanyl-aminopeptidase; ATP6AP2, prorenin/renin receptor; CMA1, chymase 1; CPA3, carboxypeptidase A3; CTSA, cathepsin A; CTSD, cathepsin D; CTSG, cathepsin G; CYP11A1, cytochrome P450 family 11 subfamily A polypeptide 1; CYP11B1, cortisolsynthase; CYP11B2, aldosteronesynthase; CYP17A1, cytochrome P450 family 17 subfamily A polypeptide 1; CYP21A2, cytochrome P450 enzyme family 21 subfamily A polypeptide 2; DPP3, dipeptidyl-peptidase 3; ENPEP, glutamylaminopeptidase (aminopeptidase A); GR, glucocorticoidreceptor; HSD11B1, hydroxysteroid (11-beta) dehydrogenase 1; HSD11B2, hydroxysteroid (11-beta) dehydrogenase 2; IGF2R, insulin-like growth factor 2 receptor; KLK1, tissuekallikrein; LNPEP, leucyl/cystinylaminopeptidase; MAS1, MAS1 proto-oncogene; MME, membranemetallo-endopeptidase; MR, mineralocorticoidreceptor; NLN, neurolysin (metallopeptidase M3 family); PREP, prolylendopeptidase; REN, renin; RNPEP, arginylaminopeptidase (aminopeptidase B); THOP1, thimetoligopeptidase 1. Images of IGF2R36, ATP6AP237, MR38, GR39, G-protein coupled receptors (AGTR1, AGTR2, GPER, and MAS1) 40 and LNPEP41 (19).

Local BM RAS has significant effects on hematopoietic systems, particularly on myeloid, and erythroid cells (3, 12, 19). Local BM RAS is involved in the regulation of important peptides that control hematopoiesis. With the help of ACE, Ang I transformed into Ang II, while bioactive SP, Ac-SDKP and Ag 1-7 were inactivated by ACE. Also, during this process, substance P (SP) is secreted from nerve endings. BM stromal and hematopoietic cells secrete RAS peptides by the AT1 and NK1 receptors that coordinate the effect of Ang II and SP, respectively as depicted in Figure 1. Additionally, it has been proven that important receptors of Ang 1-7 and MAS are available in the BM stroma (38). The BM microenvironment contains Mas receptors, which control the proliferative role of Ang 1-7 on HSCs. Ang 1-7 is created from Ang-II or Ang-I with the help of ACE2. Hematopoietic recovery after myelosuppression increases with Angiotensin (1-7)(5, 17). Besides all these, RAS plays a role in the pathogenesis of various diseases (42).

RAS is an extremely complex system consisting of a series of enzymes, peptides and receptors known to play a role in the formation of hypertension and atherosclerosis (43). It has been shown that most of the enzymes and peptides described initially as a hormonal system of RAS can be made locally in various organs, including blood vessels (43). RAS has a critical function in the management of blood flow, fluid volume, blood pressure and electrolyte balance (6). The overworking of RAS participates in the pathogenesis of various clinical situations, such as the beginning and progression of atherosclerosis (6). Ang II is the peptide known as the main last effector molecule of the classical RAS line, consisting of ACE, Ang II and angiotensin receptor type I (AT1R) (43). Ang II is produced in the brain, kidney and blood vessels. Ang II triggers hypertension through transcription activation, reactive oxygen species (ROS) production, inflammation and numerous alternative cellular events (44, 45). Ang II takes part in all stages of the pathogenesis, up to early lesion formation, growth, progression and plaque rupture, and as a result causes atherosclerosis (44, 45). Oxidative stress and inflammation make up the majority of the mechanism of action of Ang II on atherosclerosis (46). Inflammatory cells in atherosclerotic lesions are generally thought to originate from BM (15). Angiotensin II improves erythroid differentiation in the BM by interacting with the AT1R (15). Fukuda et al. analyzed a few BM chimeric mice whose BM cells were positive or negative for AT1R (47). They demonstrated that AT1aR in BM cells attend in the pathogenesis of atherosclerosis (47). Another study showed that AT1a receptor was expressed by human BM CD34^+^, CD38^−^ cells, and lymphocytes (48). Ang II has been reported to stimulate the differentiation of human CD34 + hematopoietic progenitors from cord blood (48).

Besides all these, atherosclerosis due to hypercholesterolemia is affected by the pharmacological antagonism of AT1 receptors or the reduction of the AT1A receptor (49). Hypercholesterolemia induces the production of angiotensin peptides for the effect of AT1A receptor deficiency on atherogenesis (49). There is also a close relation between cardiac RAS and the hematopoietic BM RAS (3, 12). Myocardial tissue healing through HSC plasticity shows the interaction between local cardiac RAS and hematopoietic RAS (23).

## Atherosclerosis and Central Nervous System

Atherosclerosis is often assessed as a chronic inflammatory disease, because inflammation has a significant function in all stages of atherosclerosis (50, 51). Studies have shown that atherosclerotic disease is often the cause of the onset of ischemic cerebro-vascular events (52–54). The atherogenic process is attended by flow-mediated inflammatory alterations in endothelial cells (EC) (55). In early-stage atherosclerosis, endothelial damage, abnormal lipid metabolism and hemodynamic damage are the causes of the disease (55). In late-stage atherosclerosis, lots of macrophages and inflammatory cytokines leak into the vascular wall, excrete matrix metalloproteinases (MMPs) and result in plaque rupture, bleeding and thrombosis (56). The harmonious effect of all proinflammatory signals on the plaque increases inflammation and also prevents the regeneration of structural elements that support the mechanical stability of the inflamed tissue (57).

Atherosclerosis is a chronic inflammatory syndrome that affects the unity and activity of major blood vessels which supplies the brain. As a result, chronic inflammation in the vessels impairs cerebral blood flow and neurovascular communication, which is important for cerebrovascular function (58). Chronic atherosclerosis has an effect on the brain throughout the life of people (59–62). Atherosclerosis leads to impaired vascular integrity, causing cognitive decline, stroke and vascular dementia (58). Atherosclerosis is most effective on large and medium-sized arteries, including internal carotid and vertebral arteries. Atherosclerosis and associated cardiovascular diseases can cause a wide variety of vascular diseases and lesions in the brain. These lesions caused by atherosclerosis occur as a result of arterial stiffness and inflammation (63). All these events take place with a complex mechanism.

There is an association between the autonomic nervous system (ANS) and BM cells. This relationship between BM stromal cells, HSCs and nerve terminals has been defined as the ‘neuro-reticular complex’ (64, 65). A distinctive feature of early hypertension is endothelial dysfunction (8). BM-derived endothelial progenitor cells (EPC) participate in the healing of damaged endothelium. Studies have shown that EPC numbers and functions are lower in patients with hypertension and cardiovascular disease (66–69).

## Central Nervous System and Hypertension

All components of RAS are found in the brain (70, 71). The first finding that Ang (1-7) could be produced in areas of the CNS was obtained from studies of the hydrolysis of [125I]-Ang I in brain homogenates (72). It has been shown as immunostaining for Ang (1-7) in the paraventricular, supraoptic and suprachiasmatic nuclei of the hypothalamus, in the stria terminalis bed nucleus, substantia innominata, median exclusivity and neurohypophysis (73), (74). Other studies have shown the effect of the immune system and neuroimmune pathways in hypertensive patients (9, 75).

Hypertension is a very significant risk factor for cardiovascular diseases. It remains a global public health problem (75). Various effects such as salt sensitivity and high systemic RAS activity are included in the pathophysiology of hypertension (76, 77). Moreover, studies have shown that disruptions in activity within the cardiovascular CNS fields as a result of increased sympathetic and reduced parasympathetic impulse to the peripheral organs cause end organ injury, vascular/endothelial dysfunction, and hormonal instability (78, 79). Recently, significant advances have been made in the treatment of hypertension using ACE inhibitors or AT1 receptor blockers, diuretics, α-adrenoreceptor antagonists, RAS inhibitors and calcium channel blockers (75). Molecular and neuronal changes at the brainstem and hypothalamus level have been shown to contribute to neurogenic hypertension. Studies have shown that sympathetic activation not only starts hypertension but also protects it (75). The first evidence of the significance of the autonomic nervous system for BM cell homeostasis was obtained from studies demonstrating the regulation of BM cell activity (80). It was showed that the release of BM HSPCs is rhythmically regulated in a circadian manner, for which sympathetic drive is essential (80–82).

## Local Bone Marrow Renin Angiotensin System and Central Nervous System Within the Context of Essential Hypertension

BM has an essential function in hematopoiesis regulation (19). BM HSPCs are in communication with cells of secondary lymphoid organs, such as the spleen, which control HSPC differentiation and maturation (83, 84). The relationship of BM and HSPCs with hypertension has recently attracted interest. It is thought to be a bidirectional brain-BM communication hypothesis, and this hypothesis is based on various evidences (7, 9).

Ang II related HT lead to a recommendation that activation of microglia in the pre-sympathetic cardio-regulatory brain fields may precede both the activation of the sympathetic drive and the increase in blood pressure (9, 75). Prohypertensive signals such as increased Ang II cause neurovascular-glial inflammation in the cardio-regulator areas of the brain (7). Dysfunctional ANS output is characterized by increment in the sympathetic and a reduction in the parasympathetic impulse to the periphery, including BM (7). This process induces an increment in peripheral Ang II (7). Thus, it results in a permanent increment in the inflammatory cells and a decrease in the endothelial progenitor cells (7). Increased inflammatory cells contribute to vascular and tissue damage, while reduced endothelial progenitor cells contribute to repair of this harm, leading to cardiorenal pathology (7). Extravasation of inflammatory cells into pre-sympathetic brain areas and increased somatic afferent input from BM to the brain contribute to neurovascular-glial inflammation (7). So, it directs the dysfunctional ANS output to the environment. These pathways are circumstances that cause cardiovascular and kidney pathophysiology and resistant hypertension (7). On the other hand, in Ang II-induced hypertension, BM-induced AT1R receptors limit mononuclear cell aggregation in the kidney. Thus, it reduces the chronic hypertensive response, possibly through the arrangement of vasoactive cytokines (85). Dysfunctional ANS output is characterized by a sympathetic increase and a decrease in parasympathetic impulses and is involved in the pathophysiology of hypertension (7, 9).

One study demonstrated that a dysfunctional BM ANS is correlated with imbalanced EPCs and inflammatory cells in hypertension (80). They demonstrated that in their study presympathetic neuronal activation in a spontaneously hypertensive rat was related with an accelerated retrograde transfer of the gren fluorescent protein–labeled pseudorabies virus from the BM in mangasese-enhanced MRI (80). Pro-hypertensive markers, such as increased brain and systemic RAS, cause neurovascular-glial inflammation in the brain cardio-regulator sites (27). All this evidence supports the bidirectional brain-BM interaction hypothesis for cardiovascular system homeostasis and hypertension. The effect of somatic afferent input from the BM to the CNS continues to be investigated (27).

BM has an important role in neurogenic hypertension. Memory T cells are present in the BM. T-cell activation has a significant function in hypertension (9). The BM is the primary source of EPC, which play a significant function in endothelial repair in arterial or renal injury (86, 87). Chronic elevation in BM norepinephrine may disrupt the role of EPCs and this may be important in the terms of hypertension. These interactions strengthen the connection between the CNS and BM (9). Cross-communication between ANS and BM vasculature may be an important mechanism of the pathophysiology of hypertension. NE has a vasoconstrictor role in the BM and plays a significant function in controlling blood flow (9, 88). Prohypertensive signals such as Ang II cause neuro-vascular-glial inflammation in the cardio-regulator sites of the brain (7–9). Dysfunctional ANS output is represented by an increase in sympathetic and a reduction in parasympathetic impulses to the environment, including BM. As a result of these events, there is a permanent increase in inflammatory cells and a reduce in EPCs. As a result, these increased inflammatory cells cause vascular and tissue damage, while the reduction of endothelial progenitor cells causes a reduction in repair of this damage, leading to cardio-renal pathologies (7, 9). The combination of increased somatic afferent input from BM to the brain through the extravasation of inflammatory cells into pre-sympathetic brain areas and activation of TRPV1 channels contributes to neuro-vascular-glial inflammation (89–91). All these processes sustain the emerging cardiovascular and kidney pathophysiology and resistant hypertension (7).

## New Pharmacological Approaches for RAS

There are lots of described important therapeutic usages for Angiotensin (1-7) and analogs on treating cardiovascular diseases and atherosclerosis. Some studies showed new pharmacological approaches. One of them investigated a BM-specific adrenergic beta 1 and beta 2 knock out mouse chimera (AdrB1.B2 KO) to research how sympathetic impulse to the bone influences transcripts and miRNAs in the hypothalamic paraventricular nucleus (92). The results showed that there are molecular axes involved in neural-immune interactions that can serve as targets of therapeutic treatment for a dysfunctional ANS (92). Ahmari et al. produced a mouse chimera in which the BM was irradiated and exactly reconstituted with BM from beta 1 and 2 adrenergic receptor KO mice (93). The study showed that genetic ablation of beta 1 and 2 adrenergic receptors in the BM directs an impressive modification in the BM immune system mediators. This results in decreased circulation levels of a subtype of T cells, neutrophils and macrophages (93). To research Ang-(1-7)-dependent Mas receptor function, Yang et al. used apoE-KO and apoE/Mas-KO mice with Ang-(1-7) or saline for 6 weeks (94). To check whether Ang-(1-7) regulates atherosclerosis through a NO-dependent pathway, apoE-KO mice were used with the NO synthase inhibitor in the presence or lack of Ang-(1-7) (94). Ang-(1-7) has been shown to have protective vascular effects through Mas receptor activation (94).

## Future Prospects and Hypotheses

In light of all these data, it is thought that the autonomous control of the BM has an important function in hypertension and there is bidirectional communication between the brain and the BM in this procedure (7). It is thought that there will be important developments that will provide new therapeutic targets in the therapy of hypertension with new studies in this area. It is obvious that there will be innovations at genetic level in the treatment of hypertension with the clarification of this process. Angiotensin peptides on HSCs can be edited with clustered regularly interspaced short palindromic repeats (CRISPR). Cellular therapy and gene edition can contribute to the treatment of essential hypertension. It can change the biology of HSCs that will go to the CNS in an anti-atherogenic and anti-hypertensive direction by genomic edition with CRISPR, by down-regulating AT1 receptors on the HSCs and upregulating MAS receptors. Another therapeutic target will be active microglia, which can affect the activity of neurons in the cardio-regulatory regions of the brain, which can control BM and effect the role of BM HSPCs (7). In addition to the known effects of RAS in many areas, future research and clinical studies are required to explain the different roles of local tissue RAS, including local BM RAS, and to use them as therapeutic targets.

## Author Contributions

RC was responsible for the writing of the article. IH served as scientific adviser, drafted the article, and revised it critically for important intellectual content. All authors contributed to the article and approved the submitted version.

## Conflict of Interest

The authors declare that the research was conducted in the absence of any commercial or financial relationships that could be construed as a potential conflict of interest.
